# The Comparison of Endovascular and Open Surgical Treatment for Ruptured Abdominal Aortic Aneurysm in Terms of Safety and Efficacy on the Basis of a Single-Center 30-Year Experience

**DOI:** 10.3390/jcm12227186

**Published:** 2023-11-20

**Authors:** Mansur Duran, Amir Arnautovic, Cem Kilic, Julian-Dario Rembe, Joscha Mulorz, Hubert Schelzig, Markus Udo Wagenhäuser, Waseem Garabet

**Affiliations:** 1 Department of Vascular and Endovascular Surgery, Marienhospital Gelsenkirchen, Teaching Hospital of Ruhr-University Bochum, Virchowstraße 135, 45886 Gelsenkirchen, Germany; m.duran@st-augustinus.eu; 2 Department of Vascular and Endovascular Surgery, University Hospital of Dusseldorf, Moorenstr. 5, 40225 Düsseldorf, Germany; amir.arnautovic@med.uni-duesseldorf.de (A.A.); julian-dario.rembe@med.uni-duesseldorf.de (J.-D.R.); hubert.schelzig@med.uni-duesseldorf.de (H.S.); waseem.garabet@med.uni-duesseldorf.de (W.G.); 3 Department of Vascular and Endovascular Surgery, KLINIKUM Westfalen GmbH, Am Knappschaftskrankenhaus 1, 44309 Dortmund, Germany; kilicce@gmx.de

**Keywords:** ruptured abdominal aortic aneurysm, open surgical repair, endovascular aortic repair, patient survival

## Abstract

Objective: Ruptured abdominal aortic aneurysm (rAAA) is a critical condition with a high mortality rate. Over the years, endovascular aortic repair (EVAR) has evolved as a viable treatment option in addition to open repair (OR). The primary objective of this study was to compare the safety and efficacy of EVAR and OR for the treatment of rAAA based on a comprehensive analysis of our single-centre 30-year experience. Methods: Patients treated for rAAA at the Department of Vascular and Endovascular Surgery, University Hospital Düsseldorf, Germany from 1 January 1993 to 31 December 2022 were included. Relevant information was retrieved from archived medical records. Patient survival and surgery-related complications were analysed. Results: None of the patient-specific markers, emergency department-associated parameters, and co-morbidities were associated with patient survival. The 30-day and in-hospital mortality was higher in the OR group vs. in the EVAR group (50% vs. 8.7% and 57.1% vs. 13%, respectively). OR was associated with more frequent occurrence of more severe complications when compared to EVAR. Overall patient survival was 56 ± 5% at 12 months post-surgery (52 ± 6% for OR vs. 73 ± 11% for EVAR, respectively) (*p* < 0.05). Patients ≥70 years of age showed poorer survival in the OR group, with a 12-month survival of 42 ± 7% vs. 70 ± 10% for patients <70 years of age (*p* < 0.05). In the EVAR group, this age-related survival advantage was not found (12-month survival: ≥70 years: 67 ± 14%, <70 years: 86 ± 13%). Gender-specific survival was similar regardless of the applied method of care. Conclusion: OR was associated with more severe complications in our study. EVAR initially outperformed OR for rAAA regarding patient survival while re-interventions following EVAR negatively affect survival in the long-term. Elderly patients should be treated with EVAR. Gender does not seem to have a significant impact on survival.

## 1. Introduction

Abdominal aortic aneurysm (AAA) is characterised by localised dilation exceeding 1.5 times its normal size, with a higher prevalence in men (approximately 5% of males and 1% of females aged 60 and older) [1]. Gender disparities in AAA outcomes, in particular the worse results for females, warrant investigation [2].

Ruptured AAA (rAAA) involves acute haemorrhage from the AAA, a life-threatening vascular emergency requiring immediate intervention due to the substantial mortality risk. It is the most feared sequala of AAA, with 32% of patients dying prior to hospital admission, and is associated with a high overall mortality rate of up to 81% [3]. Even with post-surgical treatment, follow-up mortality can reach 42% [4], with outcomes varying between treatment options [5]. 

Historically, open repair (OR) has been the mainstay of treatment for rAAA. This approach involves surgical exposure of the aorta and graft application to exclude the ruptured segment, but it carries significant perioperative risks, especially in elderly or medically compromised patients [6]. 

Over the past few decades, endovascular aortic repair (EVAR) has revolutionised rAAA management. EVAR avoids the extensive abdominal incision and has become the most frequently applied treatment. This entails deploying endovascular stent grafts through small groin artery incisions, effectively excluding the ruptured aorta segment. EVAR offers shorter procedural times, reduced 30-day mortality rates (particularly in high-risk patients), and lower perioperative risk [7,8], as well as improved cost-effectiveness and quality of life during the follow-up [9,10]. This approach extends candidacy to elderly patients who were previously considered unfit for surgery, reducing perioperative mortality [11].

However, the adoption of EVAR in the treatment of rAAA has raised questions about its long-term durability and effectiveness, especially in an aging population with multiple co-morbidities [12]. 

Balancing the short-term advantages of EVAR with the need for sustained, durable results in these critically ill patients remains a clinical challenge.

Despite recent advances in perioperative care and surgical techniques, the optimal management of rAAA remains a subject of ongoing research and clinical debate. This study’s primary objective was to comprehensively analyse our single-centre 30-year experience to compare the safety and efficacy of EVAR and OR for rAAA treatment.

## 2. Materials and Methods

Patients treated for rAAA at the University Hospital Düsseldorf, Germany Department for Vascular and Endovascular Surgery were included in this retrospective observational cohort study. The study period was from 1 January 1993 to 31 December 2022 for OR-treated rAAA patients and from 1 January 2013 to 31 December 2022 for EVAR-treated rAAA patients. All relevant data were retrieved from achieved medical records. Inclusion criteria were age >18 years, retro- or intraperitoneal haemorrhage from AAA on initial computed tomography angiography (CTA) scan, and available follow-up data. The patient cohort was sub-categorised into three decades for survival rate estimation. Such decades were defined as follows: 1 January 1993 to 31 December 2002; 1 January 2003 to 31 December 2012; and 1 January 2013 to 31 December 2022. Only within the last decade, patients treated with EVAR were also included. 

Statistical analysis was performed using IBM SPSS Statistics, version 25 (IBM, Armonk, NY, USA). Data are shown as the median ± SD and min-max range for ratio scale data or absolute frequency with percentage (%) for nominal scale data. Logistic regression analysis (log-likelihood ratio test) was used to examine whether the patient mortality rate is associated with patient characteristics, baseline emergency data at the time of admission, and patient co-morbidities. Where logistic regression was not feasible, ‘-’ was stated. Results are presented with 95% confidence intervals (odds ratios).

**Figure 1:**
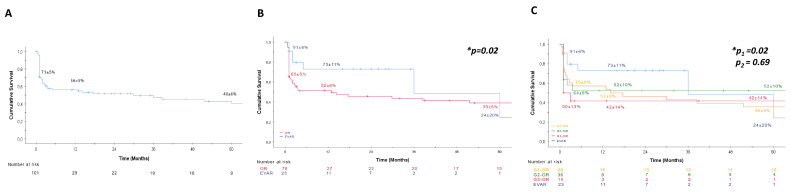
Kaplan–Meier estimator for patient survival. (**A**) Kaplan–Meier estimator for overall patient survival for the entire study cohort. 30-day,12-month and 5-year patient survival rates are depicted. (**B**) Kaplan–Meier estimator for patient survival following open repair (OR) and endovascular aortic repair (EVAR) for ruptured abdominal aortic aneurysm (rAAA). EVAR was found to have an early benefit for patient survival vs. OR, which disappears during the follow-up. 30-day, 12-month and 5-year patient survival rates are depicted. (**C**) Kaplan–Meier estimator for patient survival following EVAR or OR from 1993–2002 (G1-OR), 2003–2012 (G2-OR) or 2013–2022 (G3-OR). No difference was found for patient survival following OR between the different decades (p_2_), while EVAR was found to have an early survival benefit. 30-day, 12-month, and 5-year patient survival rates are depicted for all subgroups (p_1_). Breslow test was applied. * *p* < 0.05.

**Figure 2:**
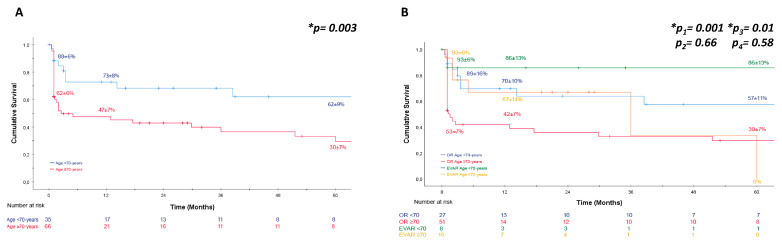
Kaplan–Meier estimator for patient survival for different age groups. (**A**) Kaplan–Meier estimator for patient survival by age group in the setting of OR or EVAR treatment for rAAA. Patient survival was better in the <70 age group vs. ≥70 age group regardless of the surgical care performed. 30-day, 12-month and 5-year patient survival rates are depicted. (**B**) Kaplan–Meier estimator for patient survival of those <70 and ≥70 years of age, respectively, for surgical care performed. The OR group showed better patient survival in the <70 age group vs. ≥70 age group (p_1_); this difference was not detectable in the EVAR group for the same sub-groups (p_2_). In the cohort of patients ≥70 years of age, there was a survival advantage for patients treated by EVAR vs. OR (p_3_); this advantage was not shown for patients <70 years of age for the same sub-groups (p_4_). 30-day, 12-month, and 5-year patient survival rates are depicted. The Breslow test was applied. * *p* < 0.05.

**Figure 3:**
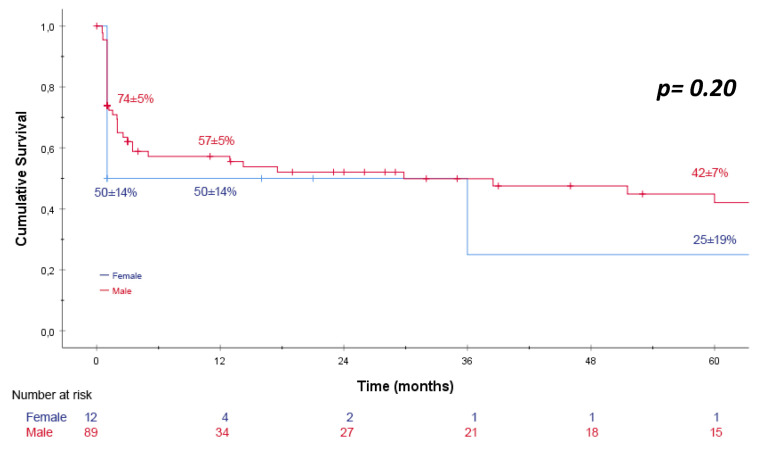
Kaplan–Meier estimator for sex-specific patient survival following open repair (OR) or endovascular aortic repair (EVAR) for ruptured abdominal aortic aneurysm (rAAA). The Kaplan–Meier estimator showed no sex differences in patient survival for the overall cohort regardless of the method used. 30-day, 12-month and 5-year patient survival rates are depicted. The Breslow test was applied.

Survival rates were estimated using the Kaplan–Meier method for overall patients (Figure 1), different age groups (Figure 2), and sex-specific patients (Figure 3). The Gehan–Breslow–Wilcoxon test was applied to compare survival rates for endoleak and re-intervention following endovascular repair. The chi-squared test with Fisher’s exact test and Student’s *t*-test were used to determine the association between peri- and/or post-operative complications and 30-day mortality. Logistic regression analysis (log-likelihood ratio test) was used to examine whether the patient mortality rate is associated with established risk factors for rAAA. An α-error of 5% was accepted; therefore, a significance level of *p* < 0.05 was used. 

The study was approved by the institutional ethical review board of the Heinrich-Heine-University Duesseldorf (study number: 4634).

## 3. Results

The investigated cohort consisted of 101 patients with complete data sets at a mean age of 74.2 ± 10.2 years with 89 males (88.1%) and 12 females (11.9%).

Patient characteristics, baseline data at the time of admission to the emergency room (ER), and co-morbidities were analysed for the OR and EVAR groups only for the last decade from 1 January 2013 to 31 December 2022, as reliable data were only available for this time period. The same applies to the analysis of differences for procedurally specific data and for postoperative complications between the OR and the EVAR group.

### 3.1. Patient Characteristics/Emergency Room/Co-Morbidities

The data for these endpoints were analysed for any association with patient survival. Within the last decade, a total of 37 cases (28 males, 9 females) with rAAA were treated, using OR in 14 cases (38%) and EVAR in 23 cases (62%). The median age of the cohort was 70.07 ± 9.1 years in the OR group and 76.78 ± 10.2 in the EVAR group. The median AAA diameter was 78.9 ± 20.4 mm in the OR group and 67.7 ± 23.0 mm in the EVAR group. Interestingly, no association with patient survival was observed in either the OR or EVAR groups (Table 1).

At the time of admission to the emergency room, the median heart rate of patients was 95.6 ± 17.8 beats/min in the OR and 96.8 ± 21.3 beats/min in the EVAR group. Of note, there was a need for preoperative blood transfusion (red blood cell concentrates) in both study populations with a median of 18.3 ± 18.1 transfusions in the OR group, while patients in the EVAR group only received a median of 7.5 ± 8.7 blood transfusions. Again, we did not observe any association with patient survival for either the OR or EVAR groups (Table 1). 

The most prevalent co-morbidity in the patient cohort was arterial hypertension (aHT), while only a minority of patients had a history of myocardial infarction or suffered from type 2 diabetes mellitus (T2DM). Of note, we did not find an association with patient survival for any co-morbidity in either the OR or the EVAR groups (Table 1).

### 3.2. Procedural Parameters

In the next step, we compared the procedural parameters between the OR and the EVAR groups. Again, this analysis was conducted for the last decade only as reliable data were only available for this period. 

As expected, the median procedure time was longer in the OR (242.4 ± 81.8 min) vs. the EVAR group (178.4 ± 121.2 min). Interestingly, the median blood loss was 3700 ± 2462 mL in the OR group and 915 ± 827 mL in the EVAR group; however, this difference was not statistically significant. The length of intensive care unit (ICU) stay was 6.1 ± 5.4 days in the OR group and 6.5 ± 6.2 days in the EVAR group. Also, no statistically significant difference was found between the study groups for this endpoint. When analysing patient 30-day mortality, a noteworthy difference was observed as seven patients in the OR group died within 30 days after the operation, while only two patients died in the EVAR group in the same period (*p* < 0.05). A similar outcome can be reported for in-hospital mortality. Here, eight patients from the OR group died compared to three deaths in the EVAR group (*p* < 0.05). The median in-hospital stay was 14.5 ± 23.8 days in the OR group and 16.6 ± 13.6 days in the EVAR group (Table 2).

### 3.3. Postoperative Complications

Clavien–Dindo classification was used to categorise and analyse postoperative complications for both the OR and EVAR groups. Severe grade V complications, involving cardiac arrest, pulmonary embolism, acute mesenteric ischemia, bleeding, splenectomy due to bleeding, pneumonia, and sepsis, were observed more frequently in the OR vs. the EVAR group (*p* < 0.05). In contrast, grade I complications occurred more often in the EVAR than the OR group, which illustrates the difference in invasiveness between both treatments (Table 3).

We further investigated whether endoleak (EL) rates were associated with patient mortality in the EVAR group. Here, we observed a total of 8 ELs: 5 type II EL and 3 type I or III Els, respectively. The presence of EL itself was not associated with increased mortality regardless of the EL-type. However, when re-intervention was necessary, this was associated with increased mortality (*p* < 0.05) (Table 4).

### 3.4. Patient Survival

Patient survival analysis included the period from 1 January 1993 to 31 December 2022 for OR-treated rAAA patients and from 1 January 2013 to 31 December 2022 for EVAR-treated rAAA patients.

Overall patient survival rate regardless of the applied treatment was 71 ± 5% at 30-days, 56 ± 5% at 12 months, and 40 ± 6% at five years post-surgery (Figure 1A). When procedure-specific survival is considered, we observed a 30-day survival of 65 ± 5% for OR and 91 ± 6% for EVAR; the 12-month survival was 52 ± 6% for OR and 73 ± 11% for EVAR, and the 5-year survival was 39 ± 6% for OR and 24 ± 20% for EVAR (*p* < 0.05) (Figure 1B). In summary, our data show an early survival advantage for the EVAR group that reverses during follow-up. Interestingly, there was no difference in terms of the survival of patients with rAAA when comparing the last three decades for OR (Figure 1C).

Next, we examined the dependence of patient survival on age. We found that patients over 70 years of age had worse survival than patients under 70 years of age regardless of the treatment method. This was shown to be the case at 30 days as well as at 12 months and 5 years (*p* < 0.05) (Figure 2A). However, this age-related survival benefit existed only for the OR group (<70 years: 30-day survival: 89 ± 10%, 12-month survival: 70 ± 10%, 5-year survival: 57 ± 11%; ≥70 years: 30-day survival: 53 ± 7%, 12-month survival: 42 ± 7%, 5-year survival: 30 ± 7%) (*p* < 0.05); it was not detectable in the EVAR group (<70 years: 30-day survival: 93 ± 6%, 12-month survival: 86 ± 13%, 5-year survival: 86 ± 13%; ≥70 years: 30-day survival: 93 ± 6%, 12-month survival: 67 ± 14%) (Figure 2B). Considering age groups rather than the applied treatment method, we observed a survival advantage for EVAR vs. OR in patients ≥70 years of age (*p* < 0.05) (Figure 2B). In the group of patients <70 years of age, there was no survival benefit for either treatment method.

Looking at the gender-specific outcome, there was a similar patient survival rate for females vs. males regardless of the surgical treatment applied (females: 30-day 50 ± 14%, 12-month 50 ± 14%, 5-year 25 ± 19%, males: 30-day 74 ± 5%, 12-month 57 ± 5%, 5-year 42 ± 7%) (Figure 3). 

## 4. Discussion

The current retrospective study investigated the comparative outcome between EVAR and OR for the treatment of rAAA in a German high-volume centre over a multidecadal period. In summary, the study found reduced in-hospital mortality for EVAR-treated patients. In addition, EVAR patients were less likely to have more severe complications. Furthermore, the current study suggests an early survival advantage for EVAR, which vanished in further follow-up. With regard to survival, older patients seem to benefit in particular from EVAR vs. OR, while gender did not seem to affect outcome, regardless of the surgical treatment method.

The optimal treatment for rAAA has been extensively studied in the literature. Here, mortality is of utmost interest. Notably, a collaboration-based pooled analysis of three prospective randomised clinical trials reported a 1-year survival rate of 38.6% for the EVAR and 42.8% for the OR group, with a pooled odds ratio of 0.84 [13]. Of note, the early survival advantage of EVAR vs. OR may be pronounced in haemodynamically unstable rAAA patients [14]. A more recent meta-analysis that included almost 7000 patients reported similar findings, with a lower perioperative mortality after EVAR, although the hazard of overall mortality during follow-up was lower [15]. 

In the context of surgical risks, the meta-analyses conducted by Kontopodis et al. (2020) and Wang et al. (2020) provide valuable insights. These studies comprehensively analysed a substantial body of data to assess the comparative surgical risks associated with different treatment modalities for patients with rAAA. Both meta-analyses collectively support the notion that EVAR, in general, presents reduced surgical risks compared to OR [16,17]. In another metanalysis, which incorporated three randomized controlled trials and 22 observational studies with a total of 31,383 patients, it was observed that EVAR maintained its benefits in long-term mortality when compared to open repair for ruptured abdominal aortic aneurysms [18].

The reported mortality rates of the current study are similar. Interestingly, we also found that mortality after OR did not change significantly, even over a multidecadal period, which means that further reductions in mortality are likely to be based primarily on further evolutions in endovascular therapy or perioperative management. Despite the advantages of EVAR in the short-term follow-up, EVAR does not come without a cost.

It has been shown that the re-intervention rate following EVAR during long-term follow-up is as high as 19% at 5 years and 35% at 14 years, even in an electively cared-for patient cohort [19]. Given that subsequent interventions following both EVAR and OR have been linked to a more than ten-fold increase in postoperative mortality, it underscores the importance of reducing complications associated with re-intervention [20]. The results of our study point in the same direction, as we found that reinterventions in EVAR-treated patients were associated with increased mortality. Given that reinterventions and associated ELs seem to be important, it is crucial to identify parameters associated with an increased likelihood of developing an EL. To this end, Çetinkaya et al. defined morphological features of the AAA neck structure such as a length less than 15 mm, a diameter of more than 28 mm, and a conical and/or calcified structure to be associated with an increased likelihood of type 1a EL development [21]. Also, a patent IMA was associated with the highest risk of type 2 EL development following EVAR [22]. However, it should be noted that a preoperative assessment of EL probability based on these parameters is unlikely to have any influence on the treatment decision in an emergency situation.

During the last few years, there has been increasing emphasis on the benefits of centralised care for AAA, which also has major relevance for rAAA treatment. In this context, a comprehensive nationwide registry analysis conducted by Sawang et al. explored the connection between surgical volume and perioperative mortality in cases of non-elective AAA repair. Their results revealed an inverse relationship between the overall hospital caseload but not the individual surgeon’s caseload and perioperative mortality in the context of non-elective AAA repair. Of interest, the effect was observed within the emergency OR group, while the authors did not observe a volume-related mortality association in the case of EVAR [23]. Similar experiences have been reported by others; however, the survival benefit of centralised treatment of OR for rAAA was found to be particularly dependent on the positioning of the proximal clamp and vanished when controlled for this parameter [24]. Since instances necessitating supraceliac clamping typically pertain to situations where patients are experiencing haemodynamic instability, it seems that a high level of technical proficiency and pooled specialised interdisciplinary expertise is critical for patient survival in such scenarios.

Age is a major determinant of outcome in many areas of modern medicine and is also a key influencing factor in rAAA disease. In particular, a series of 125 patients demonstrated that advanced age serves as an independent predictor of in-hospital mortality in rAAA [25]. 

Age-specific differences in various physiological parameters, which could partially be attributed to reduced activity levels, may contribute to the observed outcome differences. Specifically, increased systolic blood pressure (BP) and elevated total peripheral resistance (TPR) concomitant with a reduction in cardiac parameters such as left ventricular end-diastolic volume (LVEDV) and left ventricular mass (LVM) can be noted [26,27]. In addition, arterial stiffening occurs with age and is closely associated with the progression of cardiovascular disease [28]. 

In particular, age over 75 years seems to be a risk factor for perioperative mortality, although the survival rates even in octogenarians seem encouraging; therefore the decision for therapy should not only depend on age but should also take the co-morbidity profile into account [29,30]. Considering the results of our study, EVAR can be recommended as the primary therapy choice for patients over 70 years of age with regard to the probability of survival.

AAA disease has some gender-specific features. The prevalence is four times higher in men over 65 than and ranges from 1.7–4.5% in men versus 0.5–1.3% in women [31,32]. The rate of diameter expansion is higher in women compared to men and the likelihood of fatal rupture is three times as high in women [33,34]. Of particular note, the data regarding outcomes for women after rAAA repair are inconsistent in the literature. While some authors report no gender-specific differences in mortality, others found a higher 30-day mortality and a lower likelihood of discharge at home in women [35,36]. Li et al. also reported a higher perioperative and 8-year mortality rate following both EVAR and OR for women compared with men, underlining the need for an in-depth evaluation of these disparities, aiming to improve AAA care for women [37]. However, we did not find any differences between female and male survival in rAAA with regard to the treatment chosen.

This study has major limitations. Due to the small group size and the monocentric study setup, the generalisability of the results is difficult. This is particularly true in the context of gender-specific variations. The retrospective design may have heavily biased the results obtained. The incomplete clinical documentation for cases from the early decades makes interdecadal comparison somewhat difficult. While EVAR under local anaesthesia is preferred for the treatment of rAAA, it is important to note that some patients arrived intubated by the emergency physician, which could have influenced the outcome.

## 5. Conclusions

In conclusion, our results show an early survival advantage of patients treated by EVAR over the OR group. Elderly patients with rAAA should be treated by EVAR whenever possible and gender does not seem to have a significant impact on survival. The need for reintervention remains the major issue of EVAR, affecting survival in the long-term follow-up.

## Figures and Tables

**Table 1:** Association between baseline/emergency room (ER) parameters and co-morbidities and mortality for ruptured abdominal aortic aneurysm (rAAA). Data are shown as mean ± SD and min-max range for ratio scale data or absolute frequency with percentage (%) for nominal scale data. Data are presented for the open repair (OR) and endovascular aortic repair (EVAR) cohort separately. Logistic regression analysis (log-likelihood ratio test) was applied to examine whether patient mortality is associated with various essential parameters associated with rAAA. Where logistic regression was not feasible, ‘-‘ was stated. Results are presented with 95% confidence intervals (CI).

	Open Repair (OR) Median ± SD (Min–Max) or Absolute Frequency (%) (*n* = 14)	Odds Ratio (95% CI)	*p* Value	Endovascular Aortic Repair (EVAR) Mean ± SD (Min–Max) or Absolute Frequency (%) (*n* = 23)	Odds Ratio (95% CI)	*p* Value
Baseline parameters						
Age (years)	70.07 ± 9.1 (56–85)	1.045 (0.925, 1.199)	0.481	76.78 ± 10.2 (58–93)	1.078 (0.980, 1.215)	0.128
Gender, female (*n*)	4 (28.6)	0.333 (0.014, 3.718)	0.383	5 (21.7)	0.577 (0.073, 4.550)	0.606
max. AAA diameter, (mm)	78.9 ± 20.4 (45–123)	1.014 (0.960, 1.083)	0.615	67.7 ± 23.0 (22–130)	1.020 (0.980, 1.069)	0.340
Emergency room (ER)						
Unconsciousness in ER (*n*)	3 (21.4)	-	-	5 (21.7)	0.500 (0.023, 4.408)	0.555
Heart rate in the ER (beats/min)	95.6 ± 17.8 (66–121)	0.992 (0.923, 1.063)	0.808	96.8 ± 21.3 (59–135)	0.985 (0.937, 1.031)	0.507
Preoperative systolic blood pressure, (mmHg)	118 ± 27.9 (80–165)	1.009 (0.966, 1.061)	0.674	120.1 ± 27.3 (85–170)	0.968 (0.918, 1.006)	0.106
Preoperative blood transfusion, (*n*)	18.3 ± 18.1 (1–67)	1.081 (0.992, 1.264)	0.087	7.5 ± 8.7	1.040 (0.934, 1.174)	0.451
Serum creatinine (mg/dL)	1.3 (0.64–2.4)	6.495 (0.526, 312.0)	0.155	1.3 ± 1.1 (0.4–5.75)	0.808 (0.157, 1.955)	0.669
Co-morbidities						
History of myocardial Infarction	1 (7.1)	-	-	1 (4.3)	-	-
CHD	4 (28.6)	0.667 (0.057, 7.548)	0.733	6 (26.1)	3.250 (0.449, 25.10)	0.237
PAOD	3 (21.4)	1.667 (0.121, 42.430)	0.708	5 (21.7)	0.500 (0.023, 4.408)	0.555
aHT	9 (64.3)	-	-	20 (87.0)	0.167 (0.007, 2.096)	0.162
Smoking	3 (21.4)	0.286 (0.011, 3.931)	0.348	4 (17.4)	-	-
Hyperlipidaemia	1 (7.1)	-	-	12 (52.2)	0.240 (0.028, 1.503)	0.130
Pulmonary disease	3 (21.4)	1.667 (0.121, 42.430)	0.708	6 (26.1)	1.200 (0.135, 8.559)	0.858
Cerebrovascular disease	2 (14.3)	-	-	4 (17.4)	-	-
History of stroke or transient ischemic attack	1 (7.1)	-	-	4 (17.4)	-	-
CKD w/o dialysis	3 (21.4)	1.667 (0.121, 42.430)	0.708	5 (21.7)	5.250 (0.659, 52.510)	0.116
T2DM	1 (7.1)	-	-	2 (8.7)	-	-
Treatment-specific parameter						
Local anaesthesia (*n*)	-^#^	-^#^		10 (43.5)	0.964 (0.160, 5.795)	0.968

max.: maximum, ER: emergency room, CHD: coronary heart disease, PAOD: peripheral arterial occlusive disease, aHT: arterial hypertension, CKD: chronic kidney disease, w/o: without, T2DM: type 2 diabetes mellitus. #—no local anaesthesia applied in the case of open aortic repair.

**Table 2:** Procedural parameters. Data are shown as mean ± SD and min-max range for ratio scale data or absolute frequency (*n*) with percentage (%). Data are presented for open repair (OR) and endovascular aortic repair (EVAR) for ruptured abdominal aortic aneurysm (rAAA). Data were analysed using Student’s *t*-test and Fisher’s exact test. Significant findings are presented in bold.

	Open Repair (OR) (*n* = 14)	Endovascular Aortic Repair (EVAR) (*n* = 23)	*p* Value
Operation time (min)	242.4 ± 81.8 (132–458)	178.4 ± 121.2 (69–510)	0.09
Blood loss in (mL),	3700 ± 2462 (1300–6800)	915 ± 827 (330–1500)	0.184
ICU stay (days)	6.1 ± 5.4 (0–15)	6.5 ± 6.2 (0–19)	0.824
30-day mortality (*n*)	7 (50.0)	2 (8.7)	**0.023**
In-hospital mortality (*n*)	8 (57.1)	3 (13.0)	**0.008**
In-hospital stay (days)	14.5 ± 23.8 (1–95)	16.6 ± 13.6 (1–48)	0.738

**Table 3:** Post-operative complications. Data are presented for open repair (OR) and endovascular aortic repair (EVAR) for ruptured abdominal aortic aneurysm (rAAA). Data are presented as frequency distribution and percentages. Clavien–Dindo classification was used to categorise postoperative complications. Complications could be assigned to the individual stages several times based on severity. Data were analysed using Fisher’s exact test. Significant findings are presented in bold.

	Open Repair (OR) (*n* = 14)	Endovascular Aortic Repair (EVAR) (*n* = 18)	*p* Value
Overall	10 (71.4)	18 (78.3)	
Grade I (*n*)	0	6 (26.1)	0.65
		Type II endoleak (*n* = 3), respiratory insufficiency (*n* = 1), lymph fistula (*n* = 1), myocardial infarction (*n* = 1), tachyarrhythmia (*n* = 1), aortic syndrome with aortic intramural haematoma (*n* = 1)	
Grade II (*n*)	1 (7.1)	2 (8.7)	1.0
	Nosocomial pneumonia	Lymph fistula (*n* = 1), bradyarrhythmia (*n* = 1), cardiac arrhythmia (*n* = 1), acute renal failure (*n* = 1)	
Grade III (*n*)	0	3 (13.0)	0.275
		Lymph fistula (*n* = 1), wound infection (*n* = 2), bleeding femoral artery (*n* = 1), type II endoleak (*n* = 1), pleural effusion (*n* = 1), retroperitoneal haematoma (*n* = 1)	
Grade IV (*n*)	2 (14.3)	4 (17.4)	1.0
	Respiratory insufficiency with pneumonia (*n* = 2), tracheotomy (*n* = 1), acute renal failure (*n* = 1), burst abdomen (*n* = 1)	Acute renal failure (*n* = 2), dialysis (*n* = 2), myocardial infarction (*n* = 1), type Ia endoleak with revision and OR (*n* = 1), respiratory insufficiency with pneumonia (*n* = 2), arrhythmia (*n* = 1), tracheotomy (*n* = 1)	
Grade V (*n*)	7 (50)	3 (13.0)	**0.023**
	Cardiac arrest (*n* = 1), pulmonary embolism (*n* = 1), acute mesenteric ischemia (*n* = 2), bleeding (*n* = 2), splenectomy because of bleeding (*n* = 1), pneumonia (*n* = 1), sepsis (*n* = 3). acute limb ischemia (*n* = 1), leriche syndrome (*n* = 1), multiple-organ dysfunction (*n* = 5), respiratory insufficiency (*n* = 2), dialysis (*n* = 2)	Reintervention (*n* = 3), bypass (*n* = 1), limb ischemia (*n* = 1), respiratory insufficiency (*n* = 3), acute renal failure (*n* = 3), multi-organ dysfunction (*n* = 3), type 1 endoleak (*n* = 1), type III endoleak, rupture iliac artery (*n* = 1), bleeding (*n* = 2), infection of retroperitoneal haematoma (*n* = 1), spondylodiscitis (*n* = 1)	

**Table 4:** Endoleak (EL) and re-intervention following endovascular aortic repair (EVAR) for ruptured abdominal aortic aneurysm (rAAA). Data are presented as absolute frequency with percentages. Data were analysed using Cox regression to evaluate the association of each parameter with patient survival. Results are presented with the 95% confidence interval (CI). Significant findings are presented in bold.

Type of Endoleak	Endovascular Aortic Repair (EVAR) (*n* = 23)	Odds Ratio (95% CI) (Cox Regression)	*p*-Value
No EL (*n*)	15 (65.2)	0.392 (0.086, 1.779)	0.225
EL (*n*)	8 (34.8)	2.553 (0.562, 11.599)	0.225
Type II EL (*n*)	5 (21.7)	1.140 (0.209, 6.208)	0.880
Type I and type III EL (*n*)	3 (13.0)	4.883 (0.809, 29.473)	0.084
Re-intervention (*n*)	7 (30.4)	10.608 (1.156, 97.335)	**0.037**

## Data Availability

Data is contained within the article.
